# Biological and Cellular Effects of Percutaneous Electrolysis: A Systematic Review

**DOI:** 10.3390/biomedicines12122818

**Published:** 2024-12-12

**Authors:** Jacobo Rodríguez-Sanz, Sergi Rodríguez-Rodríguez, Carlos López-de-Celis, Miguel Malo-Urriés, Soledad Pérez-Amodio, Román Pérez-Antoñanzas, Sergio Borrella-Andrés, Isabel Albarova-Corral, Miguel Ángel Mateos-Timoneda

**Affiliations:** 1 Faculty of Medicine and Health Sciences, International University of Catalonia, 08028 Barcelona, Spain; jrodriguezs@uic.es (J.R.-S.); srodriguezr@uic.es (S.R.-R.); 2 ACTIUM Functional Anatomy Group, International University of Catalonia, 08028 Barcelona, Spain; 3 Fundació Institut, Universitari per a La Recerca a l’Atenció, Primària de Salut Jordi Gol i Gurina (IDIAPJGol), 08028 Barcelona, Spain; 4 Department of Physiatry and Nursery, Health Sciences Faculty, University of Zaragoza, 50009 Zaragoza, Spain; malom@unizar.es (M.M.-U.); sergiocai04@gmail.com (S.B.-A.); ialbarova@unizar.es (I.A.-C.); 5 Department of Bioengineering, Universitat International de Catalunya, 08028 Barcelona, Spain; sperezam@uic.es (S.P.-A.); rperezan@uic.es (R.P.-A.); mamateos@uic.es (M.Á.M.-T.); 6 Bioengineering Institute of Technology, Universitat International de Catalunya, 08028 Barcelona, Spain

**Keywords:** percutaneous electrolysis, cell marker, biological effects, invasive physiotherapy, healing process, inflammation

## Abstract

**Background**: Percutaneous electrolysis is an invasive physical therapy technique that is receiving attention. The objective of this article is to evaluate the biological and cellular effects of percutaneous electrolysis and its influence on tissue healing processes. **Methods**. The search strategy performed in PubMed, Cochrane Library, and Web of Sciences databases resulted in a total of 25 studies. Once inclusion and exclusion criteria were applied, seven studies were finally included in this systematic review. The biological effects of percutaneous electrolysis were evaluated and grouped into pro-inflammatory and anti-inflammatory effects, cell death, and extracellular matrix and tissue remodeling effects. **Results**. Percutaneous electrolysis generates a significant pro-inflammatory increase in the chronic tendon condition of *IL1β-6-18-1α-1rn*, *NLRP3*, and M1 polymorphonuclear cells and increased expression of *COX2*, *TNFα*, *Cxcl10*, and *TGFβ1* during the first 7 days. This inflammation is regulated as of day 13. A significant increase in cell death markers, such as LDH, Yo-Pro, cytochrome C, and Smac/Diablo markers, was observed during the first 7 days. Finally, a significant increase in markers *Mmp9*, *VEGF*, *VEGFR*, PPAR-γ/tubulin, and *COL-I* was observed in the extracellular matrix and tissue remodeling, and a decrease in *COL-III* was observed during the first 7 days. In the acute inflammatory injury condition, an increase in anti-inflammatory markers, such as *IL-10-13*, *CCL1*, and IkB, and a significant decrease in pro-inflammatory cytokines, such as *IL-6*-1β, *CCL3-4-5*, *CCR5-8*, *NFkB*, and *TNFα*, were observed during the first 7 days. Finally, a significant increase in *VEGF*, *VEGFR*, and PPAR-γ/tubulin markers in the extracellular matrix and tissue remodeling was observed for this condition during the first 7 days. **Conclusions**. Percutaneous electrolysis generates a controlled local pro-inflammatory effect in chronic conditions and regulates inflammation in inflammatory injuries (during the first 7 days). Electrolysis has short-term effects (0–7 days post) of cell death and controlled extracellular matrix destruction. Additionally, it facilitates subsequent healing by improving extracellular matrix synthesis starting from 7 days after application.

## 1. Introduction

Percutaneous electrolysis consists of the application of a galvanic electrical current delivered to the targeted tissue through a solid needle under ultrasound guidance [1]. The electrical current through the needle, which is directed to the target area of the patient’s injury, and the needle itself are defined as the negative pole (cathode) [1]. The positive pole (anode) is an electrotherapy patch that is placed on the patient’s skin [1]. Electrolysis is defined as a process in which water (H2O) and sodium chloride (NaCl), present in tissues, are decomposed into their constituent chemical elements and recombine to form new substances, namely, sodium hydroxide (NaOH), hydrogen (H), and chloride (Cl), because of the passage of a galvanic current [2,3]. These processes suggest that the technique may exert effects at the cellular level.

During the application of this technique, percutaneous electrolysis may increase the partial pressure of oxygen or induce pH alterations, thereby promoting phagocytosis and immune activity in the treated area [2,4]. These reactions can cause local pH changes due to the accumulation of hydroxide ions (OH^−^) at the cathode, creating an alkaline environment, and hydrogen ions (H⁺) at the anode, resulting in an acidic environment [2,4,5]. This pH gradient could disrupt cellular homeostasis, affecting the extracellular matrix and surrounding proteins. Additionally, electrolysis produces reactive species, such as hypochlorite (ClO^−^) and hypochlorous acid (HClO), which possess antimicrobial and denaturing properties [2,4,5]. These effects, combined with the changes in the chemical microenvironment, trigger a local inflammatory response characterized by the release of inflammatory mediators and the attraction of immune cells to the treated site [6]. The induction of a local inflammatory response could facilitate the tissue repair process and is associated with the production of NaOH as a consequence of electrolysis [2,6]. While several authors support the theory that percutaneous electrolysis generates short-term local inflammation and activates pro-inflammatory cytokines [6,7], other studies suggest potential anti-inflammatory effects in the medium- to long-term through the activation of anti-inflammatory cytokines [8,9,10]. Hence, there remains no consensus on the exact mechanism of action of this technique. This lack of consensus could be due to the different injury conditions (chronic or acute) in which the technique has been applied.

Furthermore, electrolysis induces an electrochemical reaction that directly affects the cells within the targeted tissue. Evidence suggests that it triggers cellular apoptosis through the activation of caspases and the release of Smac/Diablo proteins from the mitochondria into the cytosol [11,12]. This process is mediated by the electrolytic reaction, which leads to DNA damage and binding to the CD95 receptor [13], further promoting apoptosis [11]. Consequently, this apoptotic response facilitates the healing of damaged tissues by eliminating dysfunctional cells and allowing for a more efficient regeneration process [6,11]. This cellular apoptosis is closely related to pro-inflammatory markers, such as *IL-6* [6], *TNFα* [6,11], cytochrome C [11], and Smac/DIABLO [11], as they play a key role in regulating the balance between inflammation and cell death. TNFα can directly activate the extrinsic apoptotic pathway through death receptors (*TNFR1*), while *IL-6*, although predominantly pro-inflammatory, can modulate genes associated with apoptosis in stress environments [6,11]. On the other hand, cytochrome C and Smac/DIABLO, released from mitochondria in response to cellular damage, facilitate caspase activation by overcoming apoptosis inhibitors [11]. These interactions highlight how local inflammation and mitochondrial events are interconnected in programmed cell death processes, particularly in contexts of tissue damage and repair, such as those induced by techniques like percutaneous electrolysis. Apoptosis induced by percutaneous electrolysis may contribute to tissue healing by selectively removing damaged or dysfunctional cells, preventing their accumulation, and fostering a healing environment [6,11]. This process releases pro-regenerative signals that attract macrophages and other immune cells, promoting the phagocytosis of cellular debris and the release of growth factors, such as *TGF-β* and *VEGF*, which are essential for tissue repair [6,7,8,11]. Additionally, it facilitates the transition to an anti-inflammatory environment [8,9]. This healing facilitation appears to be associated, too, with increased synthesis of the extracellular matrix by resident cells within the tissue (such as tendon cells [6,7] or muscle cells [9]), rather than with increased cellular proliferation [6,7]. However, discrepancies remain regarding these effects on the extracellular matrix and tissue remodeling. Despite these inconsistencies, most studies report a degradative effect on certain extracellular matrix components and an enhancement of endothelial growth [6,7,8,9,11].

Previous systematic reviews and meta-analyses have demonstrated the clinical efficacy of percutaneous electrolysis in reducing pain and improving function [14,15,16,17,18,19]. However, to date, no systematic review has comprehensively analyzed the biological and cellular effects of this technique or drawn definitive conclusions regarding its structural effects on tissues. Although the evidence is scarce, it may be a good starting point to draw some preliminary conclusions and further investigate the biological effects of this technique. Therefore, the objective of this review is to evaluate, through an analysis of the existing scientific literature, the biological and cellular effects of percutaneous electrolysis and its influence on tissue healing processes according to the condition (chronic or acute) of the injury.

## 2. Materials and Methods

### 2.1. Protocol and Registration

A systematic review was performed according to the Preferred Reporting Items for Systematic Reviews and Meta-Analyses (PRISMA) statement checklist. The systematic review protocol was registered in INPLASY database on 18 September 2024 with ID: 202490079 (https://inplasy.com/inplasy-2024-9-0079/, accessed on 18 September 2024).

### 2.2. Information Sources and Search

The search strategy was developed following the PICO (population, intervention, comparison, outcomes) strategy. The target population was any living organism; the intervention was percutaneous electrolysis through a solid needle; sham and/or control groups were the comparators; and the main outcome was the effects of the technique on cellular and biological markers. Due to the novelty of the technique investigated, no other filters were added. The keywords used to develop the search strategy are shown in Table 1.

The databases used in this systematic review were PubMed, Cochrane Library, and Web of Science. Furthermore, the reference lists of the included studies were reviewed to find studies that met the inclusion criteria. The final search was performed on 20 September 2024. The complete database search strategy is shown in Table 2. Moreover, we included manual searches through the reference lists of the reviewed studies and reviewed other similar systematic reviews for possible studies that might meet the inclusion criteria.

### 2.3. Eligibility Criteria and Study Selection

The inclusion criteria for the studies included in this systematic review were as follows: (1) any study that performed post-intervention measurements with percutaneous electrolysis (clinical trials, case-control studies, or quasi-experimental studies); (2) any living organism (rats, mice, cells, or humans); (3) biological and cellular marker analysis; and (4) English or Spanish language. Studies were excluded if (1) no galvanic current through a needle was used, (2) cellular and/or biological marker analysis was not performed or (3) there was failure to provide quantitative data of the results. Moreover, if studies provided data with bar charts, the corresponding author of the article was contacted to request the mean and standard deviation of each outcome.

The titles and abstracts of all the initial studies were screened by two independent authors (JRS and SRR). In case of discrepancy, a third author (CLdC) was consulted. Cohen’s Kappa index was used to assess inter-rater agreement.

### 2.4. Data Collection Process

The following data were extracted for studies included in this systematic review: (1) author’s last name and year of publication; (2) sample population; (3) treatment groups; (4) intervention characteristics (duration, dosage, intensity, frequency of application); (5) treated tissue; (6) follow-ups; (7) biological marker findings.

### 2.5. Outcomes

The primary outcome of this systematic review was to describe the expression of cytokines, apoptotic proteins, and genes related to extracellular matrix and tissue remodeling generated by percutaneous electrolysis. In addition, as secondary results, other effects found will be described.

### 2.6. Risk of Bias of Individual Studies

For the analysis of animal studies, the SYRCLE risk of bias tool [20] was used (Review Manager v5.4.1). This tool is based on the Cochrane Collaboration’s tool for assessing risk bias in randomized controlled trials [21] and is related to six types of bias: selection bias, performance bias, detection bias, attrition bias, reporting bias, and other sources of bias. Studies that performed interventions were evaluated. For human studies, the Physiotherapy Evidence Database (PEDro) scale was chosen [22]. However, as we will see in the results, this scale was not used because no human studies exist that met the criteria of the present review or measured any biological effects of percutaneous electrolysis.

Bias assessments were carried out by two reviewers (JRS and SRR), and any discrepancies were resolved after discussion.

## 3. Results

### 3.1. Search Strategy

The search strategy found a total of 25 studies (PubMed: 6; Web of Sciences: 14; Cochrane Library: 4; manual search: 1). Eighteen studies were initially included after verifying for duplicates.

### 3.2. Study Selection

**Figure 1:**
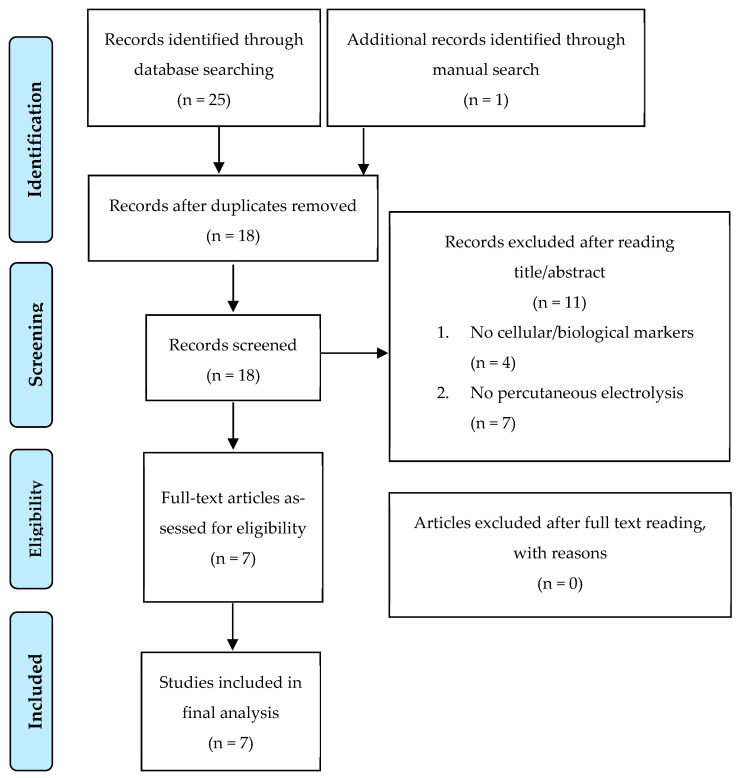
PRISMA flow chart.

We then screened by title, abstract, full text, and inclusion criteria and excluded 11 articles. The reasons for excluding these 11 studies were as follows: not measuring cellular or biological markers (*n* = 4) and not performing interventions with percutaneous electrolysis through a needle (*n* = 7). Cohen’s Kappa index showed “very good” concordance (k = 0.87) among the evaluators. A detailed selection of the studies and reasons for excluded articles are available in the PRISMA flow chart (Figure 1). Finally, seven studies were included in the final analysis.

The studies included in this systematic review had a mean score of 5.75 in the National Institutes of Health (NIH) Quality Assessment Tool for Observational Cohort and Cross-sectional Studies [23,24]. The total score ranged from 4 to 8. However, the total maximum score by study was 11 due to the cross-sectional design of all the studies included. 

### 3.3. Risk of Bias of Individual Studies

**Figure 2:**
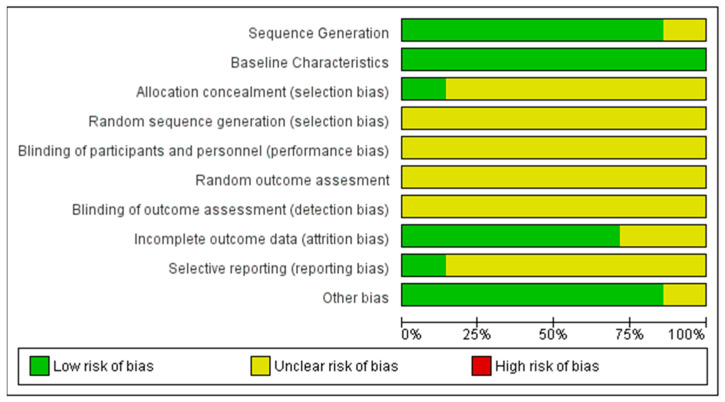
Risk of bias graph: review of authors’ judgements about each risk of bias item presented as percentages across all included studies.

**Figure 3:**
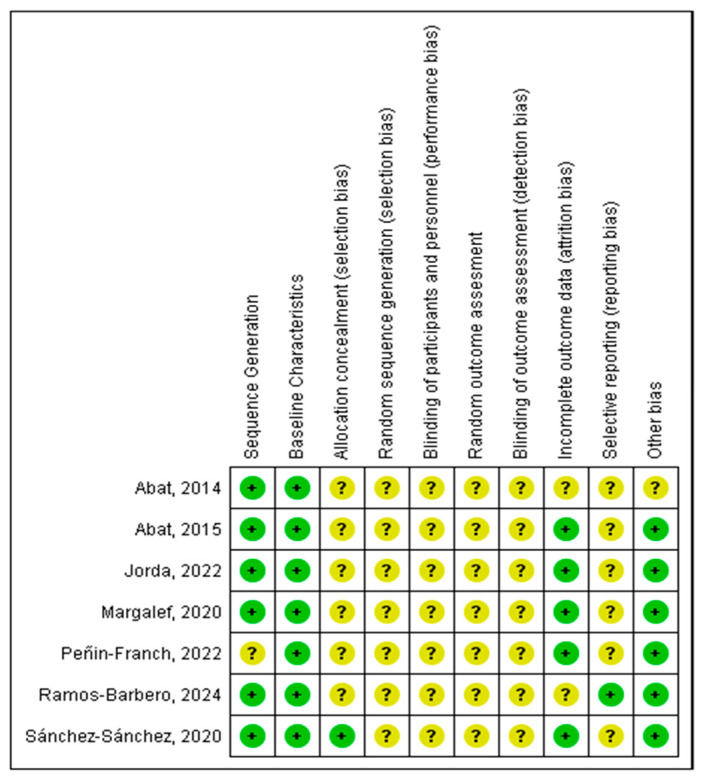
Risk of bias summary: review of authors’ judgements about each risk of bias item for each included study [5,6,7,8,9,10,11]. “+”: Low risk of bias. “?”: Unclear risk of bias.

Figure 2 and Figure 3 summarize the results of risk of bias assessment for the seven studies, using SYRCLE’s risk of bias tool for animal studies (Systematic Review Centre for Laboratory animal Experimentation). The SYRCLE risk of bias tool does not use a standardized numerical cut-off value, as its design is based on qualitative criteria [20]. However, during the review conducted by the two investigators, the following cut-off values were determined to describe the risk of bias. If 70% of the items obtained a positive “yes” response, the risk of bias was considered low. If >50% of the items were answered “no”, the risk was considered high, and if >50% of the items were answered “unclear”, the risk was considered unclear. In the present systematic review, at general levels, the studies present an unclear risk of bias. All the studies except Peñin-Franch [6] clearly describe how the group assignment sequence was applied. All the studies described the baseline characteristics of the animals. Except for the study by Sánchez-Sánchez [7], none of the studies analyzed describe the concealment of group assignment. None of the studies indicated whether the animals were randomly married, but all the papers specified the housing environment. We did not find a description of the blinding of the evaluators nor the process of randomly evaluating the results in any study. The results of the data for the entire literature were completed, and no selective reporting was evaluated.

### 3.4. Study Characteristics

**Figure 4:**
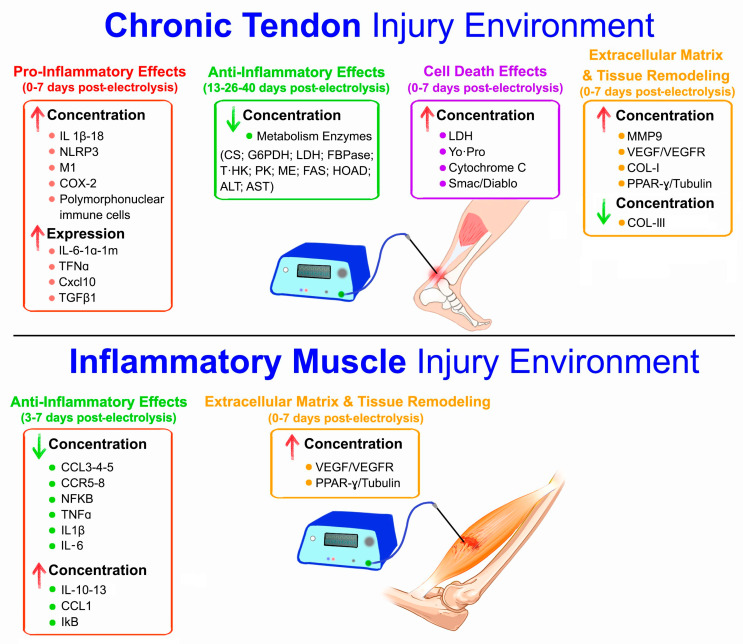
Diagram of the effects of percutaneous electrolysis depending on the environment of chronic tendon or acute muscle injury. Abbreviations: IL-6-10-13-18-1β-1α, interleukin-6-10-13-18-1β-1α; NLRP3, NOD-like receptor family pyrin domain containing 3; IL1rn, interleukin-1 receptor antagonist; TNFα, tumor necrosis factor alpha; Cxcl10, C–X–C motif chemokine ligand 10; TGFβ1, transforming growth factor beta 1; COX-2, cyclooxygenase-2; LDH, lactate dehydrogenase; Yo-Pro, Yo-Pro-1 (apoptosis marker); M1, macrophage phenotype 1; COL-I-III, collagen type I-III; Mmp9, matrix metalloproteinase 9; VEGF, vascular endothelial growth factor; VEGFR, vascular endothelial growth factor receptor; CCL1-3-4-5, C–C motif chemokine ligand 1-3-4-5; CCR5-8, C–C motif chemokine receptor 5-8; NFkB, nuclear factor kappa B; IkB, inhibitor of kappa B; Smac/Diablo, second mitochondria-derived activator of caspases/direct IAP-binding protein with low pI; PPAR-γ/tubulin, peroxisome proliferator-activated receptor gamma/tubulin; HK, hexokinase; PK, pyruvate kinase; FBPase, fructose-1,6-bisphosphatase; G6PDH, glucose-6-phosphate dehydrogenase; CS, citrate synthase; ME, malic enzyme; HOAD, 3-Hydroxyacyl-CoA dehydrogenase; FAS, fatty acid synthase; AST, aspartate aminotransferase; ALT, alanine aminotransferase.

All the included studies involved 114 rats/mice, and the mean age ranged from 5 weeks to 8 months. No human studies were found in which cellular markers were evaluated after the application of percutaneous electrolysis. All the included studies evaluated cellular and biological markers after the application of percutaneous electrolysis. Figure 4 visually details the summary effects of all the studies by injury condition. Table 3 details the main characteristics of the studies included, the type of application used, the analyzed markers, and the results obtained with the different applications. Finally, Table 4 shows the significant pre- and post-intervention quantitative results with percutaneous electrolysis for each study analyzed.

## 4. Discussion

The systematic review results show different effects on cellular and biological markers in animal studies (rats and mice). The data obtained from all the articles included (Table 3 and Table 4 and Figure 4) are analyzed below. The discussion of the results has been grouped according to the questions commonly raised about electrolysis.

### 4.1. Inflammatory or Anti-Inflammatory Effect?

There is currently controversy about the effects of percutaneous electrolysis. Some studies describe pro-inflammatory local effects [6,17], while other authors find that electrolysis can generate anti-inflammatory effects [8,9,10].

The studies describing effects on increased local inflammation were carried out in tendinopathies induced with collagenase [6,17]. These studies [6,17] found an increase in the concentrations of *IL1β*, *IL-18*, and *NLRP3* and the expression of other inflammatory markers, such as *IL-6*, *IL1α*, *IL18*, *IL1rn*, *Cxcl10*, or *TGFβ1*. In addition, increased pro-inflammatory M1 macrophages have been observed. However, direct activation of M2 macrophages by electrolysis has not been reported [6]. These findings were obtained in measurement periods not exceeding 7 days from the application of percutaneous electrolysis.

As for the studies that show a decrease in inflammation [8,9], all of them were carried out on muscular lesions induced by the toxin “notexin” in periods not exceeding 7 days from the therapeutic application. This toxin, coming from snake venom, generates important tissue necrosis together with powerful inflammation. In addition, another study measured metabolic enzymes and found that rats treated with electrolysis resolved inflammatory processes better in the medium-term (13–26–40 days post-percutaneous electrolysis) [10].

The observed results show that the condition (chronic tendinopathy or acute muscle injury) in which percutaneous electrolysis is applied is crucial. Living organisms maintain equilibrium (homeostasis) [25,26] in the face of any perturbation. It seems evident that in more stable conditions, such as those produced by collagenase in tendons (at least at the cytokine level), percutaneous electrolysis can increase local pro-inflammatory factors [6,7]. However, when inflammation levels are high in the environment (as in the notexin condition, like an acute injury), the body may experience a “cytokine storm” [25,26].

One possible explanation for certain studies [8,9,10] finding that electrolysis can regulate excessive inflammatory environments could be the interaction between the effects of electrolysis and the “cytokine storm” condition in acute injuries. In these situations, electrolysis would facilitate the homeostatic regulation of the organism, producing a cytokine balance [8,9]. This could be explained by different cytokines in response to percutaneous electrolysis that have a dual function (especially *IL-6-10-13*, *IL1β*, and *TGFβ1*) [8,9,10]. In summary, depending on the cellular environment of the organism where the technique is applied (acute inflammatory injury or a chronic lesion with less inflammation), the cytokines generated by electrolysis can interact with the environment to produce a pro- or anti-inflammatory effect. These results lead us to conclude that electrolysis has a dual pro- and anti-inflammatory effect depending on the cellular environment of the lesion, generating local inflammation in chronic pathologies and normalizing excessive inflammation in inflammatory pathologies. However, further research seems necessary to confirm these results.

### 4.2. Cell Proliferation or Cell Death?

The debate over whether percutaneous electrolysis induces cell proliferation or cell death is an ongoing discussion among physical therapists who use the technique. These differing opinions have emerged due to the tissue healing effects attributed to this procedure. However, cell proliferation (referring to the multiplication of cells near the injury) is a different concept from the enhancement of extracellular matrix synthesis processes by cells already present in the damaged tissue [6].

The results of the various studies reviewed carry significant implications. They indicate that percutaneous electrolysis induces cell death (cytochrome C, Smac/Diablo, Yo-Pro, and LDH) [6,11]. Furthermore, they suggest that an increase in current intensity is associated with greater cell damage [6]. Additionally, bactericidal effects have also been observed in treatments with percutaneous electrolysis (bacterial death) [3]. These findings provide valuable insights into the potential effects of percutaneous electrolysis, which can inform and guide future research and practice in the field of physical therapy.

Although this concept may appear counterproductive to tissue healing processes, this is not necessarily true. Percutaneous electrolysis is applied to the damaged areas of the tissue, and this cell death is associated with an increase in local inflammation [6,11]. This process is essential for tissue healing, as it activates the initial phase of inflammation. Percutaneous electrolysis may help eliminate damaged tissue by “cleaning” the injury region. This process can facilitate the subsequent stages of tissue healing by the organism, potentially accelerating regeneration. This effect is also linked to the cytokines generated by percutaneous electrolysis [27,28,29,30].

### 4.3. Extracellular Matrix and Tissue Remodeling

The improvement in extracellular matrix synthesis and tissue remodeling is a consensus reached by all the reviewed studies evaluating these markers [6,7,8,11].

In line with the increased cell death, other markers of damaged extracellular matrix destruction, such as metalloproteinase *Mmp9*, also rise with electrolysis [7].

After 7 days of application, percutaneous electrolysis aids the body in synthesizing the extracellular matrix more efficiently, increasing the synthesis of type I collagen and decreasing *COL-III* in tendons [6] or PPAR-γ/tubulin in muscles [8,11]. These findings align with previously discussed results confirming that percutaneous electrolysis can assist the body in small-scale tissue healing by enhancing extracellular matrix synthesis through local tissue cells. These results have also been observed macroscopically via ultrasound [6,8,31,32]. These authors noted improvements in the quantity and alignment of fibers in the injured tissue starting from 7 days of percutaneous electrolysis application.

Additionally, consistent with the local inflammatory effect of percutaneous electrolysis, markers of vascular endothelial growth (*VEGF*, *VEGFR*) [7,8,11] increase within periods of less than 7 days. The vascular endothelial growth factor facilitates tissue healing and activates the immune system in the injury area.

### 4.4. Clinical Implications

Percutaneous electrolysis can act as a regulator of inflammation. It can generate a local pro-inflammatory effect when the tissue environment has low levels of inflammation and can decrease inflammation levels when the tissue is in an environment with high levels of inflammation. Clinically, it can be a useful tool for chronic conditions to stimulate the injured area to initiate a local inflammatory process (the first phase of tissue healing), thereby facilitating the body’s healing of the tissue. In the case of an acute injury (e.g., a muscle tear), percutaneous electrolysis can mediate excessive inflammatory environments and regulate inflammation levels.

Electrolysis can help eliminate some of the damaged tissue through controlled cell death and extracellular matrix destruction. This situation will help to “clean” the injury region, giving the body better conditions for the subsequent healing process.

Finally, its effects on improving extracellular matrix synthesis and increasing blood supply to the injury site will facilitate the healing process of the target tissue. In cases where the injury is minor, this may be sufficient for cells to synthesize a matrix to regenerate the tissue. For larger injuries, it is likely necessary to combine percutaneous electrolysis with techniques that enhance cell proliferation and produce noticeable macroscopic healing. Additionally, all these biological effects translate into functional, structural, and symptomatic improvements. Different studies have shown improvements in neural conduction after applying percutaneous electrolysis in fibrotic areas [32,33], or enhancements in function [15,18,34,35,36,37,38] and symptoms in patients with different pathologies [14,15,16,17,18,19,31,36,37,38,39,40,41].

### 4.5. Limitations and Future Studies

This systematic review consolidates the available evidence and clinical applicability. However, there are several limitations. Firstly, the available evidence is very limited because percutaneous electrolysis with a needle has been used for less than 30 years. In addition, it is possible that articles in non-Latin alphabet languages (e.g., Chinese, Arabic, Russian) may not have been identified. Secondly, the evaluation of cellular and biological markers in all the available studies was conducted in animals. Although these studies provide relevant data, we cannot rule out the possibility that these effects may not be the same in humans. Thirdly, cellular biology is complex, and there can be many interactions between different proteins. The results of this systematic review are based on the markers evaluated in various studies and the existing literature on cellular biology. Therefore, we cannot exclude the possibility of other interactions depending on the environment and conditions of the studies. It is necessary to continue studying the biological and cellular effects of percutaneous electrolysis in humans, in different tissues, and under various injury conditions. Additionally, it is important to provide information on the effects of the technique on other cellular markers to better understand its impacts.

## 5. Conclusions

Percutaneous electrolysis generates a controlled local pro-inflammatory effect in chronic conditions and regulates inflammation in inflammatory injuries (during the first 7 days). Electrolysis has short-term effects (0–7 days post) of cell death and controlled extracellular matrix destruction. Additionally, it facilitates subsequent healing by improving extracellular matrix synthesis starting from 7 days after application.

## Figures and Tables

**Table 1:** Keywords used for the search strategy.

Population	Intervention	Outcomes
Rats	Percutaneous electrolysis	Physiological effects
Mice	EPI technique	Cellular response
Cell	Ultrasound-guided electrolysis	Metabolism
Human	Percutaneous needle electrolysis	Gene expression
	Percutaneous galvanic electrolysis	Protein expression
	Intratissue percutaneous electrolysis	Cytokines
		Inflammatory response
		Biological effects
		Chemokine
		Molecular effects
		Cellular effects
		Regeneration
		Cell proliferation
		Apoptosis
		Oxidative stress
		Interleukin
		Angiogenesis

**Table 2:** Search strategy.

**Pubmed Search Strategy**
((“percutaneous electrolysis” OR “EPI technique” OR “ultrasound-guided electrolysis” OR “percutaneous needle electrolysis” OR “percutaneous galvanic electrolysis” OR “Intratissue Percutaneous Electrolysis”) AND (“physiological effects”[Text Word] OR “cellular response”[Text Word] OR “Metabolism”[Text Word] OR “gene expression”[Text Word] OR “protein expression”[Text Word] OR “cytokines”[Text Word] OR “inflammatory response”[Text Word] OR “biological effects”[Text Word] OR “chemokine”[Text Word] OR “molecular effects”[Text Word] OR “cellular effects”[Text Word] OR “Regeneration”[Text Word] OR “cell proliferation”[Text Word] OR “apoptosis”[Text Word] OR “oxidative stress”[Text Word] OR “Interleukin”[Text Word] OR “angiogenesis”[Text Word]) AND (“rats”[Title/Abstract] OR “mice”[Title/Abstract] OR “cell” [Title/Abstract] OR “human”[Title/Abstract]))
**Cochrane and Web of Science Search Strategy**
(((“percutaneous electrolysis” OR “EPI technique” OR “ultrasound-guided electrolysis” OR “percutaneous needle electrolysis” OR “percutaneous galvanic electrolysis” OR “Intratissue Percutaneous Electrolysis”) AND (“physiological effects” OR “cellular response” OR “Metabolism” OR “gene expression” OR “protein expression” OR “cytokines” OR “inflammatory response” OR “biological effects” OR “chemokine” OR “molecular effects” OR “cellular effects” OR “Regeneration” OR “cell proliferation” OR “apoptosis” OR “oxidative stress” OR “Interleukin” OR “angiogenesis”) AND ((“rats” OR “mice” OR “cell” OR “human”))))

**Table 3:** Analysis of the methodology and main biological results obtained with percutaneous electrolysis treatments in the studies included in the systematic review.

Reference	Sample	Sample Gender	Age	Groups	Sample Characteristics	Percutaneous Electrolysis Intervention (miliAmperes:sec:impacts)	Treatment Frequency	Condition	Follow-Ups	Cellular and Biological Markers	Main Findings by Percutaneous Electrolysis Intervention
Peñin-Franch (2022) [6]	Mice (*n* = At least 4 “Unclear”)	Male	8–10 Weeks	-Control group -Dry needling group -Percutaneous electrolysis group	-NLRP3-deficient mice -Casp1/11-deficient mice -ASC-deficient mice (Pycard-/-) -Wild mice	3:3:3 3:6:2 3:6:8 6:6:2 6:6:8 12:6:2 12:6:8	Single	Chronic Tendon (Achilles)	3–7–14–21 days post-electrolysis	**Pro-Inflammatory Effects** (IL-6; IL18; IL1β; IL1α; TNF α; Cxcl10; NLRP3; COX-2; polymorphonuclear cells M1) **Anti-Inflammatory Effects** (IL1rn; TGF β1; Arg1; Fizz1; Mrc1; Ym1; M2) **Cell Death** (LDH; Yo-Pro; Pycard; Casp1) **Extracellular matrix and tissue remodeling** (COL-I; COL-III)	**Intervention: 12:6:2** **Pro-Inflammatory Effects** Significant increase in expression: (COX2; IL-6; TNFα). Significant increase in concentration: (IL1β; IL-18; NLRP3) **Cell Death** Significant increase in concentration: (LDH)
											**Intervention: 12:6:8** **Cell Death** Significant increase in concentration: (LDH; Yo-Pro)
											**Intervention: 6:6:8 and 3:6:8** **Cell Death** Significant increase in concentration: (Yo-Pro)
											**Intervention: 6:6:2** and **3:6:2** **Pro-Inflammatory Effects** Significant increase in concentration: (IL1β)
											**Intervention: 3:3:3** **Pro-Inflammatory Effects** Significant increase: (Polymorphonuclear cells M1) Significant increase in expression: (IL-6; IL1α; IL1β; Cxcl10; IL1rn; TGF β1) **Extracellular matrix and tissue remodeling** Significant increase in concentration: (COL-I) Significant decrease in concentration: (COL-III)
Sánchez-Sánchez (2020) [7]	Rats (*n* = 15)	Male	8 Weeks	-Control group (*n* = 3) -Collagenase-confirming group (*n* = 3) -Collagenase control group (*n* = 3) -Collagenase percutaneous electrolysis group (*n* = 3) -Collagenase needling group (*n* = 3)	Sprague Dawley rats	3:4:3	1 per week (3 weeks)	Chronic Tendon (Achilles)	7 days after the last electrolysis session	**Extracellular matrix and tissue remodeling** (COX2; Col1a1; Col3a1; Mmp2; Mmp3; Mmp9; VEGF, Scx, B-act; Gapdh; Rpl19)	**Intervention: 3:4:3** **Extracellular matrix and tissue remodeling** Significant increase: (COX2, Mmp9 y VEGF)
Jorda (2022) [9]	Rats (*n* = 20)	Female	7 Months	-Control group (*n* = 5) -Notexin group (*n* = 5) -Percutaneous electrolysis group (*n* = 5) -Notexin percutaneous electrolysis group (*n* = 5)	Wistar rats	6:5:4	2 times (at 7–11 days after Notexin)	Inflammatory Muscle (Quadriceps)	3 days after the last electrolysis session	**Pro-Inflammatory Effects** (CCL3; CCL4; CCL5; CCR5; CCR8; NFkB) **Anti-Inflammatory Effects** (IL-6; IL-13; IL-10; CCL1; IkB)	**Intervention: 6:5:4** **Anti-Inflammatory Effects** Significant increase: (IL-13; IL-10; CCL1; IkB) **Pro-Inflammatory Effects** Significant decrease: (IL-6; CCL3; CCL4; CCL5; CCR5; CCR8; NFkB)
Ramos-Barbero (2024) [10]	Rats (*n* = 24)	Male	Unclear	-Healthy control (*n* = 4) -Diseased control (*n* = 4) -Percutaneous electrolysis (*n* = 4) -Percutaneous electrolysis + hydroxytyrosol (*n* = 4) -Percutaneous electrolysis + maslinic acid (*n* = 4) -Percutaneous electrolysis with amino acids glycine and aspartate (*n* = 4).	Wistar rats	3:4:1	Single	Chronic Tendon (Achilles)	13–26–40 days post-electrolysis	**Anti-Inflammatory Effects (Metabolism Enzymes)** (HK; PK; FBPase; LDH; G6PDH; CS; ME; HOAD; FAS;GDH; AST; ALT)	**Intervention: 3:4:1** **Anti-Inflammatory Effects (Metabolism Enzymes)** Significant decrease: (CS; G6PDH; LDH; FBPase; T-HK; PK; ME; FAS; HOAD; ALT; AST)
Abat (2014) [11]	Rats (*n* = 24)	Female	7 Months	-Control group (*n* = 6) -Collagenase-confirming group (*n* = 6) -Percutaneous electrolysis group 3mA (*n* = 6) -Percutaneous electrolysis group 6 mA (*n* = 6)	Sprague-Dawley	3:4:3 6:4:3	Single	Chronic Tendon (Patellar)	3 days post-electrolysis	**Cell Death** (Cytochrome C; Smac/Diablo) **Extracellular Matrix and Tissue Remodeling** (VEGF; VEGFR; PPAR-γ/tubulin)	**Intervention: 3:4:3** and **6:4:3** **Cell Death** Significant increase: (Cytochrome C; Smac/Diablo) **Extracellular Matrix and Tissue Remodeling** Significant increase: (VEGF; VEGFR; PPAR-γ/tubulin)
Abat (2015) [8]	Rats (*n* = 24)	Unclear	Unclear	-Control group (*n* = 6) -Notexin group 7 days (*n* = 6) -Notexin group 14 days (*n* = 6) -Notexin percutaneous electrolysis group (*n* = 6)	Sprague-Dawley	3:5:4	Single	Inflammatory Muscle (Quadriceps)	7 days post-electrolysis	**Pro-Inflammatory Effects** (TNFα; IL-1B) **Extracellular Matrix and Tissue Remodeling** (VEGF; VEGFR; PPAR-γ/tubulin)	**Intervention: 3:5:4** **Pro-Inflammatory Effects** Significant decrease: (TNFα; IL-1B) **Extracellular Matrix and Tissue Remodeling** Significant increase: (VEGF; VEGFR; PPAR-γ/tubulin)
Margalef (2020) [5]	Mice (*n* = 3)	Male	5 Weeks	-Control group (*n* = 3 paws) -Percutaneous electrolysis group (*n* = 3 paws)	Unclear	3:3:3	Single	Gastrocnemius	Immediately post-electrolysis	**pH**	**Intervention: 3:3:3** No changes

Abbreviations: IL-6-10-13-18-1β-1α, interleukin-6-10-13-18-1β-1α; IL1rn, interleukin-1 receptor antagonist; TNFα, tumor necrosis factor alpha; Cxcl10, C–X–C motif chemokine ligand 10; TGFβ1, transforming growth factor beta 1; COX-2, cyclooxygenase-2; Arg1, arginase 1; Fizz1, found in inflammatory zone 1; Mrc1, mannose receptor C-type 1; Ym1, chitinase-like 3; LDH, lactate dehydrogenase; Yo-Pro, Yo-Pro-1 (apoptosis marker); Pycard, apoptosis-associated speck-like protein containing a CARD; Casp1, caspase-1; M1-2, macrophage phenotype 1-2; NLRP3, NOD-like receptor family pyrin domain containing 3; COL-I-III, collagen type I-III; Col1a1-3a1, collagen alpha-1(I) chain-alpha-1(III) chain; Mmp2-3-9, matrix metalloproteinase 2-3-9; VEGF, vascular endothelial growth factor; VEGFR, vascular endothelial growth factor receptor; Scx, scleraxis; B-act, beta-actin; Gapdh, glyceraldehyde 3-phosphate dehydrogenase; Rpl19, ribosomal protein L19; CCL1-3-4-5, C–C motif chemokine ligand 1-3-4-5; CCR5-8, C–C motif chemokine receptor 5-8; NFkB, nuclear factor kappa B; IkB, inhibitor of kappa B; Smac/Diablo, second mitochondria-derived activator of caspases/direct IAP-binding protein with low pI; PPAR-γ/tubulin, peroxisome proliferator-activated receptor gamma/tubulin; HK, hexokinase; PK, pyruvate kinase; FBPase, fructose-1,6-bisphosphatase; G6PDH, glucose-6-phosphate dehydrogenase; CS, citrate synthase; ME, malic enzyme; HOAD, 3-Hydroxyacyl-CoA dehydrogenase; FAS, fatty acid synthase; GDH, glutamate dehydrogenase; AST, aspartate aminotransferase; ALT, alanine aminotransferase.

**Table 4:** Data on significant results from the analyzed studies pre- and post-intervention with percutaneous electrolysis.

Reference	Condition	Biomarker	Pre-Intervention Mean (SD)		Post-Intervention Mean (SD)
Peñin-Franch (2022) [6]	Chronic Tendon (Achilles)	Pro-Inflammatory Effects	Intervention: 12:6:2		Intervention: 12:6:2
			COX2^2ΔCT^	0.000 (0.000)	0.012 (0.009)
			IL-6^2ΔCT^	0.000 (0.000)	0.133 (0.007)
			TNFα^2ΔCT^	0.002 (0.002)	0.005 (0.003)
			IL1β _(pg/mL)_	8.641 (2.348)	151.191 (58.308)
			IL-18 _(pg/mL)_	51.728 (22.983)	227.518 (52.719)
			NLRP3^2ΔCT^	4.530 (2.045)	65.727 (4.603)
		Cell Death	LDH (%)	3.088 (0.551)	7.850 (3.429)
		Cell Death	Intervention: 12:6:8		Intervention: 12:6:8
			LDH _(%)_	3.088 (0.551)	42.890 (13.831)
			Yo-Pro _(slope)_	18.287 (6.197)	154.703 (37.134)
		Pro-Inflammatory Effects	Intervention: 6:6:2		Intervention: 6:6:2
			IL1β _(pg/mL)_	8.641 (2.348)	59.572 (13.631)
		Cell Death	Intervention: 6:6:8		Intervention: 6:6:8
			Yo-Pro _(slope)_	18.287 (6.197)	118.782 (45.521)
		Cell Death	Intervention: 3:6:8		Intervention: 3:6:8
			Yo-Pro _(slope)_	18.287 (6.197)	67.661 (30.408)
		Pro-Inflammatory Effects	Intervention: 3:6:2		Intervention: 3:6:2
			IL1β _(pg/mL)_	8.641 (2.348)	30.522 (18.482)
		Pro-Inflammatory Effects	Intervention: 3:3:3		Intervention: 3:3:3
			Polymorphonuclear cells M1 _(nº)_	1.333 (2.016)	10.708 (9.727)
			IL-6^2ΔCT^	1.123 (0.569)	4.105 (1.551)
			IL1α^2ΔCT^	0.000 (0.000)	3.362-05 (2.569-05)
			IL1β^2ΔCT^	1.302 (1.142)	21.529 (22.760)
			Cxcl10^2ΔCT^	1.023 (0.243)	4.782 (3.336)
			IL1rn^2ΔCT^	1.479 (1.667)	4.533 (4.637)
			TGF β1_(Fold Change)_	0.158 (0.185)	1.000 (0.397)
		Extracellular matrix and tissue remodeling	COL-I _(%)_	13.719 (7.307)	26.083 (12.054)
			COL-III _(%)_	86.281 (7.307)	73.901 (12.047)
Sánchez-Sánchez (2020) [7]	Chronic Tendon (Achilles)	Extracellular matrix and tissue remodeling	Intervention: 3:4:3		Intervention: 3:4:3
			COX2^2ΔCT^	0.044 (0.050)	1.351 (0.706)
			Mmp9^2ΔCT^	0.000 (0.000)	8.564 (4.872)
			VEGF^2ΔCT^	0.109 (0.021)	2.208 (0.135)
Jorda (2022) [9]	Inflammatory Muscle (Quadriceps)	Anti-Inflammatory Effects	Intervention: 6:5:4		Intervention: 6:5:4
			IL-13 _(Pg/mL)_	11.499 (2.643)	28.569 (3.571)
			IL-10 _(Pg/mL)_	35.829 (12.964)	74.471 (12.839)
			CCL1^2ΔCT^	0.898 (0.156)	1.119 (0.117)
			IkB _(Arbitrary Units)_	0.447 (0.093)	0.670 (0.064)
		Pro-Inflammatory Effects	IL-6 _(Pg/mL)_	84.401(8.643)	67.109 (8.643)
			CCL3^2ΔCT^	2.321 (0.339)	1.770 (0.145)
			CCL4^2ΔCT^	2.497 (0.267)	1.843 (0.164)
			CCL5^2ΔCT^	1.920 (0.465)	1.310 (0.285)
			CCR5^2ΔCT^	2.684 (0.291)	1.453 (0.167)
			CCR8^2ΔCT^	1.585 (0.110)	1.174 (0.257)
			NFkB _(Arbitrary Units)_	1.190 (0.152)	0.810 (0.067)
Ramos-Barbero (2024) [10]	Chronic Tendon (Achilles)	Anti-Inflammatory Effects (Metabolism Enzymes)	Intervention: 3:4:1		Intervention: 3:4:1
			CS _(nmol/min/mg protein)_	7.840 (0.980)	4.580 (0.360)
			G6PDH _(nmol/min/mg protein)_	25.570 (1.040)	19.380 (1.740)
			LDH _(nmol/min/mg protein)_	4031.8 (269.1)	3107.1 (282.3)
			FBPase _(nmol/min/mg protein)_	41.380 (7.600)	35.910 (1.750)
			T-HK _(nmol/min/mg protein)_	2.260 (0.220)	1.850 (0.080)
			PK _(nmol/min/mg protein)_	327.57 (40.600)	283.03 (16.240)
			ME _(nmol/min/mg protein)_	3.970 (0.650)	4.700 (0.280)
			FAS _(nmol/min/mg protein)_	1.440 (0.110)	1.000 (0.040)
			HOAD _(nmol/min/mg protein)_	177.78 (10.330)	132.37 (3.700)
			ALT _(nmol/min/mg protein)_	125.10 (9.530)	113.68 (20.340)
			AST _(nmol/min/mg protein)_	1046.5 (100.50)	722.68 (49.840)
Abat (2014) [11]	Chronic Tendon (Patellar)	Cell Death	Intervention: 3:4:3		Intervention: 3:4:3
			Cytochrome C _(Relative Densitometry Unit)_	378.769 (78.842)	412.145 (60.679)
			Smac/Diablo _(Relative Densitometry Unit)_	321.424 (12.856)	1722.703 (49.281)
		Extracellular Matrix and Tissue Remodeling	VEGF _(Relative Densitometry Unit)_	19.028 (6.410)	48.155 (6.993)
			VEGFR _(Relative Densitometry Unit)_	29.250 (0.573)	85.592 (1.344)
			PPAR-γ/tubulin _(Relative Densitometry Unit)_	8.213 (1.006)	8.883 (0.939)
		Cell Death	Intervention: 6:4:3		Intervention: 6:4:3
			Cytochrome C _(Relative Densitometry Unit)_	378.769 (78.842)	563.608 (42.422)
			Smac/Diablo _(Relative Densitometry Unit)_	321.424 (12.856)	1474.160 (74.498)
		Extracellular Matrix and Tissue Remodeling	VEGF _(Relative Densitometry Unit)_	19.028 (6.410)	42.522 (12.239)
			PPAR-γ/tubulin _(Relative Densitometry Unit)_	8.213 (1.006)	13.107 (1.006)
Abat (2015) [8]	Inflammatory Muscle (Quadriceps)	Pro-Inflammatory Effects	Intervention: 3:5:4		Intervention: 3:5:4
			TNFα _(Pg/mL)_	32.800 (3.100)	16.200 (2.800)
			IL-1β _(Pg/mL)_	319.600 (13.50)	120.200 (17.700)
		Extracellular Matrix and Tissue Remodeling	VEGF _(Relative Densitometry Unit)_	51.800 (6.700)	85.035 (4.371)
			VEGFR _(Relative Densitometry Unit)_	38.500 (3.100)	60.300 (4.900)
			PPAR-γ/tubulin _(Relative Densitometry Unit)_	23.000 (1.800)	62.000 (6.100)

Abbreviations: IL-6-10-13-18-1β-1α, interleukin-6-10-13-18-1β-1α; IL1rn, interleukin-1 receptor antagonist; TNFα, tumor necrosis factor alpha; Cxcl10, C–X–C motif chemokine ligand 10; TGFβ1, transforming growth factor beta 1; COX2, cyclooxygenase-2; LDH, lactate dehydrogenase; Yo-Pro, Yo-Pro-1 (apoptosis marker); M1, macrophage phenotype 1; NLRP3, NOD-like receptor family pyrin domain containing 3; COL-I-III, collagen type I-III; Mmp9, matrix metalloproteinase-9; VEGF, vascular endothelial growth factor; VEGFR, vascular endothelial growth factor receptor; CCL1-3-4-5, C–C motif chemokine ligand 1-3-4-5; CCR5-8, C–C motif chemokine receptor 5-8; NFkB, nuclear factor kappa B; IkB, inhibitor of kappa B; Smac/Diablo, second mitochondria-derived activator of caspases/direct IAP-binding protein with low pI; PPAR-γ/tubulin, peroxisome proliferator-activated receptor gamma/tubulin; HK, hexokinase; PK, pyruvate kinase; FBPase, fructose-1,6-bisphosphatase; G6PDH, glucose-6-phosphate dehydrogenase; CS, citrate synthase; ME, malic enzyme; HOAD, 3-Hydroxyacyl-CoA dehydrogenase; FAS, fatty acid synthase; GDH, glutamate dehydrogenase; AST, aspartate aminotransferase; ALT, alanine aminotransferase; 2ΔCT, fold difference in expression normalized by the reference gene; Nmol/min/mg protein, nanomol in one minute per milligram of protein; Pg/mL, picograms per milliliter.

## Data Availability

All data are present in this study.
